# Cognitive-behavioral group therapy for women with hypoactive sexual desire: A pilot randomized study

**DOI:** 10.1016/j.clinsp.2022.100054

**Published:** 2022-07-26

**Authors:** Théo Lerner, Vicente Renato Bagnoli, Elsa Aida Gay de Pereyra, Lucivanda Pontes Fonteles, Isabel Cristina Esposito Sorpreso, José Maria Soares Júnior, Edmund Chada Baracat

**Affiliations:** Disciplina de Ginecologia, Departamento de Obstetrícia e Ginecologia, Hospital das Clínicas, Faculdade de Medicina da Universidade de São Paulo, São Paulo, SP, Brazil

**Keywords:** Group Therapy, Cognitive-behavioral Therapy, Female sexual dysfunction, Hypoactive sexual desire disorder

## Abstract

**Background:**

Hypoactive Sexual Desire Disorder (HSDD) is a very prevalent sexual problem, with limited options for treatment. Given that psychological factors are major contributors to the disorder, a therapy such as Cognitive-Behavioral Therapy (CBT) may be useful to treat HSDD.

**Objective:**

To evaluate the effects of group CBT on women with HSDD.

**Method:**

Clinical trial randomized study with 106 women diagnosed with HSDD, who were divided as follows: Group 1 (n = 53) underwent group CBT for 8-weeks, and Group 2 (n = 53), were put on a waiting list and used as a control group. Sexual function was assessed by the Female Sexual Quotient (FSQQ) at the initial interview and after 6-months. Mann Whitney test was used for group comparison. Main Outcome Measures: demographics, education, sexual history, FSQQ and its domains for sexual function assessment.

**Results:**

Both groups had similar characteristics regarding sexual response, self-image, and relationship with a partner at the initial interview. Women undergoing therapy showed significant improvement in sexual function when compared with the control group. The overall FSQQ result showed an average growth of 18.08 points (95% CI 12.87‒23.28) for the therapy group against a decrease of 0.83 points (95% CI 3.43‒1.77) for controls (p < 0.001). The five domains of the questionnaire also exhibited significant improvement in the therapy group: desire and interest (p = 0.003), foreplay (p = 0.003), excitation and tuning (p < 0.001), comfort (p < 0.001), and orgasm and satisfaction (p < 0.001).

**Conclusion:**

Group CBT was shown to be an effective tool for treating HSDD.

## Highlights

• Group cognitive-behavioral therapy (CBT) have a positive influence on women's sexuality.
• Group CBT was shown to be an effective tool for treating Hypoactive Sexual desire disorder in women.
• Group CBT can be a short-term focal intervention carried out by gynecologists specialized in sexual health.
• Group CBT can be used in a high-complexity care setting.

## Introduction

Hypoactive Sexual Desire Disorder (HSDD) is one of the most frequent sexual dysfunctions. Its prevalence is estimated to be between 8% and 19% among women,^1^, ^2^, ^3^, ^4^ thus representing an important problem in clinical practice[5,6] and negatively impacting the Quality of life (QoL).^7^, ^8^, ^9^ Therapeutic alternatives to deal with the problem are still controversial. The difficulty in objectively evaluating treatment outcomes, the discomfort of patients and health professionals in addressing the issue, insufficient time during a medical consultation to discuss sexuality issues, and the lack of skilled care services make HSDD a challenge in clinical practice.^5^^,^^6^^,^^10^^,^^11^

The DSM-5 (the fifth edition of the Diagnostic and Statistical Manual of Mental Disorders) placed sexual desire disorders with arousal dysfunctions under the name of Female Sexual Interest/Arousal Disorder (FSIAD).^12^ There are sparse data in the literature about the prevalence of FSIAD and the variables which interfere with this disorder,^11^ limiting its clinical applicability.WHO's (World Health Organization) ICD (International Classification of Diseases) 11[13] defines HSDD as a reduction in or absence of desire (spontaneous or in response to erotic stimulation) or an inability lasting several months associated with personal distress to maintain desire or interest after the onset of sexual activity,^6^^,^^14^ negatively impacting the woman or her partner's quality of life.

HSDD is related to a dynamic interaction of biological, psychological, interpersonal, and socio-cultural factors in a biopsychosocial model that must be taken into account in choosing the most appropriate therapeutic method to meet the demands of women.^14^^,^^15^

The HSDD treatment model should be multifactorial, covering physiological, psychological, relational, and socio-cultural aspects. In general, pharmacological alternatives to the treatment of HSDD are limited. To date, there are only two medications approved for use in HSDD in premenopausal women, flibanserin, and bremelanotide.^16^, ^17^, ^18^, ^19^ In postmenopausal women, testosterone may be used, but further studies are needed to determine long-term safety.^20^, ^21^, ^22^ Nonpharmacological approaches to HSDD, including psychotherapy, have shown promising results.^23^^,^^24^ Many psychotherapy techniques have proved to be valid for the treatment of HSDD, such as Masters and Johnson's sensate focus,^25^^,^^26^ couple therapy,^27^, ^28^, ^29^ mindfulness,^30^^,^^31^ and Cognitive-Behavior Therapy (CBT),^32^^,^^33^ which seem promising in the treatment of menopausal symptoms,^34^ genito-pelvic pain,^35^ and interpersonal relationship problems.^36^ However, further studies are needed, especially with respect to the treatment of HSDD.

CBT is a type of focal therapy whose main goal is to change dysfunctional beliefs and behaviors interfering with a specific problem. In the case of HSDD, the theoretical model of CBT establishes that such dysfunctional sexual beliefs act as predisposing factors by stipulating conditional rules for the activation of negative cognitive schemes. These schemes, once activated, provoke negative automatic thoughts and emotions which impair the processing of erotic stimuli and interfere negatively with desire.^32^^,^^37^ In a public health context, CBT appears to be effective; however, there are few studies on its effectiveness.^32^^,^^38^ Our study intends to evaluate the effect of CBT on a group of Brazilian women with HSDD.

## Method

### Study design and setting

This is a clinical trial study based on women who attended the Sexual Medicine Outpatient Clinic with sexual complaints.

This study was approved by the institutional Ethics Committee (case n° 0346/08-IRB) and was registered in the Brazilian Clinical Trial Registry (https://ensaiosclinicos.gov.br/) under #12087

All methods were performed in accordance with the relevant guidelines and regulations, as the checklist CONSORT.

All patients signed an informed consent form agreeing to participate in the study.

### Materials

This study was conducted with 189 women who came to the outpatient clinic with sexual complaints. Diagnosis of HSDD was reached using the diagnostic criteria of ICD-11 (absence of or marked reduction in desire or motivation to engage in sexual activity manifested by any of the following: 1) Reduced or absent spontaneous desire (sexual thoughts or fantasies); 2) Reduced or absent responsive desire for erotic stimuli or stimulation; or 3) Inability to maintain desire for or interest in sexual activity once initiated. This decrease in desire may occur episodically or continuously over a period of several months and leads to significant personal suffering).^13^

### Eligibility criteria

Women over 20-years old who met the diagnostic criteria for HSDD were included in this study.

The authors excluded women with other sexual disorders, such as orgasmic disorder or sexual pain disorder, uncompensated systemic disease (hepatic cirrhosis, Chron's disease, collagen disease, severe heart disease, multiple sclerosis), psychiatric problems (schizophrenia, bipolar disorder, severe depression), dyspareunia (endometriosis, Sjöegren's syndrome, vaginal stenosis after radiotherapy, climacteric syndrome), and use of psychoactive drugs or any other drug that impairs libido.

### Patient allocation

**Fig. 1:**
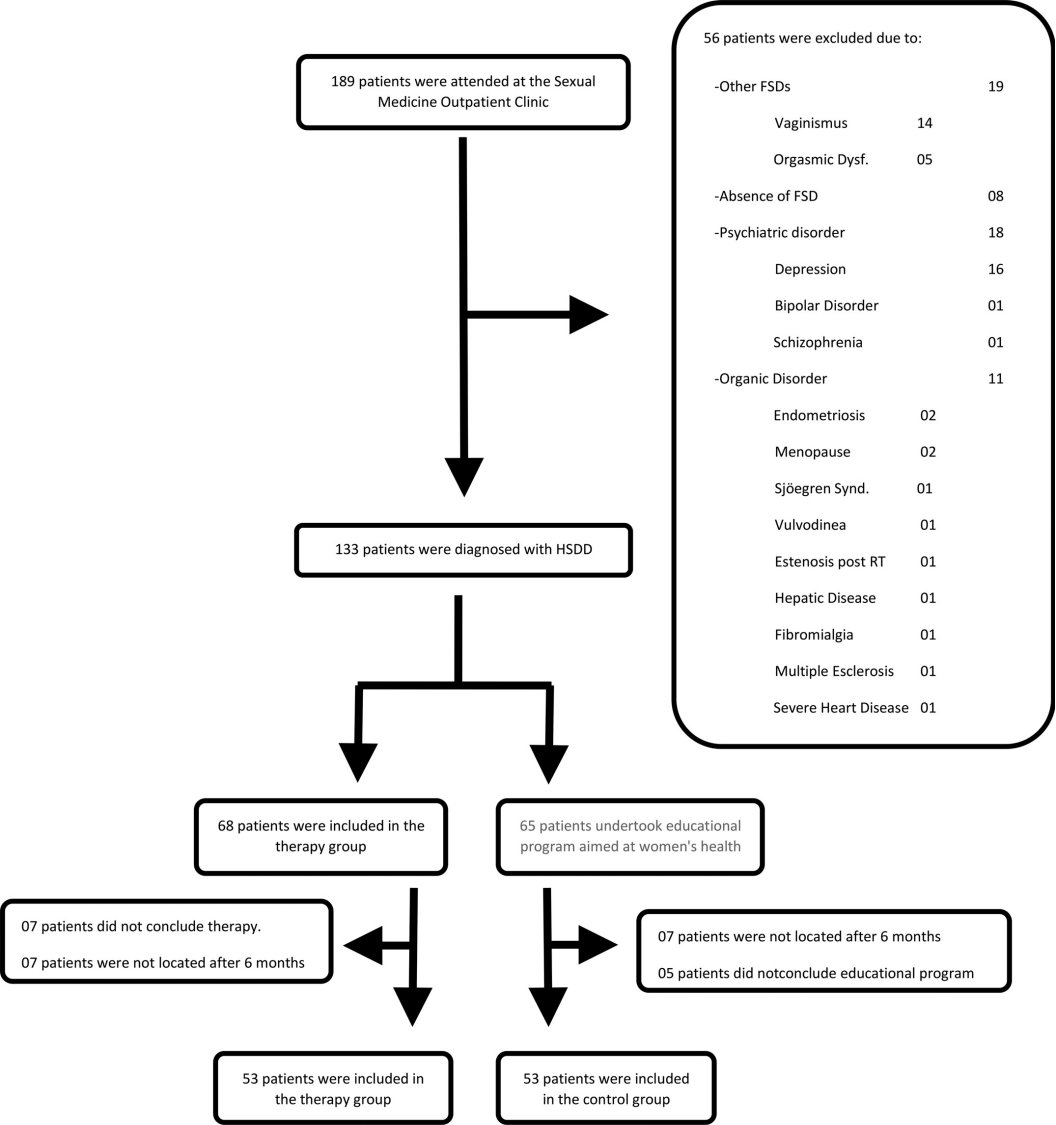
Study algorithm.

Prior to group allocation, women referred to the sexuality outpatient clinic were interviewed and examined. Data on demographics, education, sexual history, and relationships were collected. A hormone profile was requested to evaluate androgen levels. The presence of depression was assessed by the Beck inventory. After applying the eligibility criteria, 56 patients were excluded (Fig. 1). The remaining 133 patients were randomly divided into two groups using an online randomizer software (https://www.randomizer.org). A random sequence of 150 numbers was generated. The patients received a number on the list in order of entrance. Patients with odd numbers were assigned to the intervention group and patients with even numbers were assigned to the control group. Generation of random allocation sequence, enrollment of participant, and group assignment were done by the same professional and supervised by two others. There was no blinding of patients or care providers.

The 68 patients included in the intervention group were referred for group CBT, whose methodology will be described later. Fifty-three patients completed the 8 groups CBT sessions and were reevaluated after 6-months. One patient was excluded from the study because of a severe marital conflict involving domestic violence during treatment.

The 65 patients in the control group undertook an 8-week educational program aimed at women's health and cancer prevention.^39^ Fifty-three of these patients completed the educational program. Six months later they were located, and their data were used in this study.

The demographics of the intervention and control group will be presented in the results.

The algorithm for patient allocation in this study is shown in Fig. 1.

### Intervention

Patients attended an 8-session group CBT treatment. The group size was between 5 to 10 participants. The purpose of the treatment was to change the participants’ beliefs and behavior related to sexual activity. In order to address possible sources of interpretation bias in the intervention group, all groups were conducted by the same professional.

At each weekly session, patients were encouraged to discuss their thoughts and experiences relative to their sexual activity, and homework exercises were proposed to improve self-awareness and enable the identification of dysfunctional thoughts. Patients were instructed to avoid engaging in sexual intercourse with their partners during therapy, in order to minimize the influence of previous behavioral schemes such as the anticipation of negative emotional results from sexual activity.

The order of the exercises followed a semi-structured script, and it was dependent on the participants’ responses. In every exercise, patients were encouraged to perceive and record the sensations, thoughts, and emotions they had during or after the exercise.

In general terms, the homework tasks were assigned in the following order:

Session 1: Mirror – Patients were oriented to look at their whole-body image in front of a mirror, searching for positive points, identifying concrete data such as size and age, and recording associated thoughts during the exercise. The goal was to restructure their self-image and increase their self-perception.
Session 2: Bath ‒ Patients were instructed to identify their flow of thoughts while showering and to try to focus their attention on sensations at the time, identifying underlying feelings and thoughts. After the shower, the use of a skin moisturizer was suggested, and the attention focus exercise was to be repeated. The purpose of this exercise was to improve the patients’ attention and allow them to record pleasurable sensations.
Session 3: Genital Visualization – Patients were asked to try and identify concepts about their self-image and sexual roles while observing their genitals in the mirror. This exercise provided the input for discussions on anatomy and the physiology of sexual response, allowed the answers to pertinent questions, and the identification of dysfunctional beliefs.
Session 4: Genital Manipulation – Patients were required to identify and validate feelings aroused by the genitals in order to improve self-awareness.
Session 5: Sensate Focus – With their partners, the patients were asked to engage in the sensate focus exercise while avoiding contact with genitals and breasts. The purpose of this exercise was to improve the couple's communication skills and to re-establish physical intimacy without the pressure of sexual performance.
Session 6: Sensate Focus – With their partners, the patients were instructed to engage in the sensate focus exercise while allowing contact with genitals. Penetration should be avoided.
Session 7: Nondemanding intercourse – Patients engaged in sexual intercourse with allowance for penetration without the demand for reaching orgasm. The focus was to be on intimacy and awareness of feelings and needs.
Session 8: Progress evaluation/Follow-up scheduling/Free intercourse.

### Measurements

Patients were evaluated by the Female Sexual Quotient Questionnaire (FSQQ),^40^ a ten-item self-response tool, which evaluates five domains of sexuality (desire and interest, foreplay, excitement and attunement, comfort, orgasm, and satisfaction). The questionnaire was administered upon admission to the outpatient clinic (baseline) and six months after the end of the therapy group.

Variation in FSQQ scores (6-months-baseline) and its domains were used as a parameter to assess changes in sexual function.

### Statistical analysis

The Kolmogorov-Smirnov test was used for comparison of the two continuous data samples (FSQQ scores at 6-months and at baseline) of each group. In both groups, the normal distribution of data was not accepted (p < 0.05).

Score variation was reported using summary measures (mean, standard deviation, median, minimum, maximum, and 95% Confidence Interval for means) according to groups and group comparison using the Mann-Whitney test.^41^

Sample power, based on the variation of FSQQ scores,^40^^,^^41^ was calculated at 85% (β-15) with a minimum of 43 patients for each group. Predicting a 20% to 30% loss, the authors recruited 50% more women.

Cronbach's Alpha coefficients were calculated to assess the internal consistency of the FSQQ, using wessa.net software (Wessa, P. (2020), Free Statistics Software, Office for Research Development and Education, version 1.2.1, URL https://www.wessa.net/). Values above 0,7 were considered acceptable for purposes of group comparison.

## Results

Clinical and demographic data are shown in Table 1. There were no significant differences between women in the CBT group and controls with regard to ethnicity, religion, education, marital status, length of the relationship, and parity. The sexual response phases, the frequency of sexual intercourse, subjective impressions about the quality of the relationship, and body image were questioned, with similar distribution in both groups.

**Table 1:** Demographic data of the sample.

	Intervention	Control	p
Age	40.6 ± 6.36	38.5 ± 10.36	0.312
Race, n (%)			
White	26 (49%)	19 (35.8%)	
Nonwhite	27 (51%)	34 (63.2%)	
Religion, n (%)			
Catholic	34 (64.1%)	29 (54.7%)	
Evangelic	14 (26.4%)	16 (30.2%)	
Other	5 (9.5%)	8 (15.1%)	
Education, n (%)			
Elementary	29 (54.7%)	32 (60.3%)	
High school	21 (39.6%)	18 (34.0%)	
College	3 (5.7%)	3 (5.7%)	
Marital Status, n (%)			
Married	38 (71.7%)	42 (79.2%)	
Single	6 (11.3%)	5 (11.3%)	
Divorced	5 (9.5%)	6 (9.5%)	
Widow	4 (7.5%)	‒	
Relationship lenght (years)	13.63 ± 10.88	12.43 ± 9.31	0.514
Parity	1.96 (±1.49)	2 (±2.17)	0.339

Demographic data were analyzed using the Chi-Square test for proportional data and the unpaired Student *t*-test for quantitative data (age, parity, and relationship length).

Table 2 compares the variation of the FSQQ domain results between the two groups. The initial and final scores of the intervention group showed significant differences in all of the FSQQ domains, except in the foreplay domain, whereas the score variations of the control group did not present significant differences in any of the FSQQ domains.

**Table 2:** Variation in the FSQQ scores by domain.

	Intervention		Controls			Variation in FSQQ scores			
Domain	Baseline	Final	P (bxf)	Baseline	Final	P (bxf)	Controls	Intervention	p
Desire and interest^a^^,^^c^	15.64 ± 7.55	19.83 ± 6.78	0.005	11.79 ± 6.27	11.38 ± .86	NS	-0.42 ± 4.37	4.19 ± 8.18	0.043
Foreplay^a^^,^^c^	7.13 ± 3.75	8.62 ± 2.96	0.055	5.25 ± 3.56	4.87 ± 3.05	NS	-0.38 (±2.19)	1.49 ± 3.94	0.004
Excitement and attunement^b^^,^^c^	10.51±5.64	14.70 ± 5.47	0.0003	9.64 ± 6.26	9.64 ± 5.79	NS	0.00 (±2.61)	4.19 ± 5.96	<0.001
Comfort^b^^d^	11.83 ± 6.28	14.40 ± 5.05	0.02	11.79 ± 6.50	10.98 ± 6.56	NS	-0.81 (±2.99)	2.57 ± 5.73	<0.001
Orgasm and satisfaction^b^^,^^c^	7.05 ± 4.79	12.77 ± 5.91	<0.00001	5.62 ± 4.74	5.77 ± 4.90	NS	0.15 (±3.03)	5.72 ± 5.87	<0.001
FSQQ Total^a^^,^^c^	50.49 ± 19.04	68.57 ± 19.20	<0.001	42.34 ± 19.10	41.51 ± 17.54	NS	-1.45 (±9.49)	18.15 ± 19.52	<0.001

a Comparing baseline FSQQ intervention group scores with controls (p < 0.02) – Desire & Interest, Foreplay, and the total FSQQ score.

b Comparing baseline FSQQ intervention group scores with controls (NS) ‒ Excitement & Attunement, Comfort, Orgasm & Satisfaction.

c Comparing final FSQQ intervention group scores with controls (p < 0.00001) ‒ Desire & Interest, Foreplay, Excitement & Attunement, Orgasm & Satisfaction, and the total FSQQ score.

d Comparing final FSQQ intervention group scores with controls (p < 0.01) – Comfort.

**Fig. 2:**
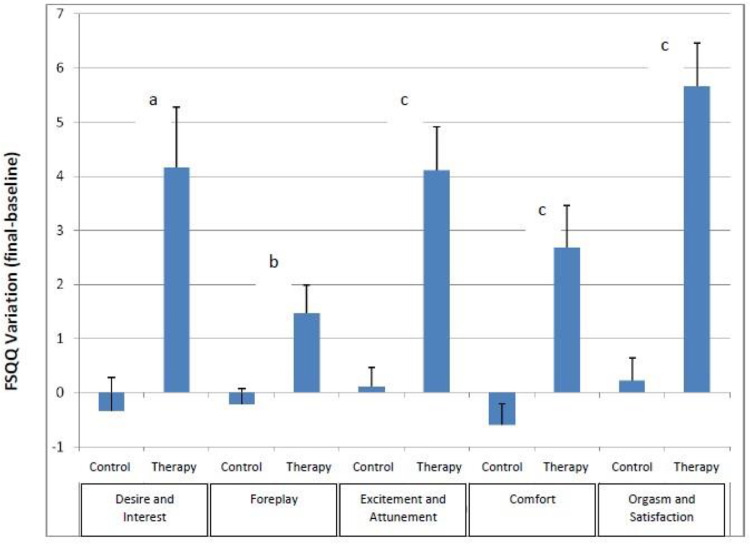
Domain according to group. Comparing the intervention group with the control group in terms of (a) Desire and interest (p < 0.05); (b) Foreplay (p < 0.01); and (c) Excitement and attunement, comfort, orgasm, and satisfaction (p < 0.001).

At the end of 6-months, the intervention group showed significant improvement in all of the FSQQ domains when compared to the control group. Improvements are shown in Fig. 2. The CBT group had a wider and statistically more significant variation in the total FSQQ scores than the controls (p < 0.001). Internal consistency for this sample was good (Cronbach's alpha = 0.83 ± 0.34) for the multi-item FSQQ in both groups. Cronbach's alpha for initial FSQQ and after 6-months were 0.76 ± 0.24 and 0.88 ± 0.48 respectively.

## Discussion

Female sexual disorders are highly prevalent, and they have a negative psychological impact on women's quality of life. However, there are few therapeutic options. Besides, sexuality is a subject that is rarely discussed by health professionals.^42^ The discomfort caused by sexual disorders (HSDD included) can lead to a significant decrease in self-image and self-esteem, interfering in effective interpersonal relationships. In our study, CBT has emerged as a seemingly effective alternative to attenuate the repercussions of HSDD.

HSDD still represents a great clinical challenge since its diagnosis and treatment are limited by several factors: a) Limited or insufficient time during the medical visit; b) Discomfort felt by both the health professionals and the patients in addressing sexuality-related issues; c) Few available specialized services; d) Few or ineffective therapeutic options; e) Absence of a single etiological factor; f) Overlap of several disorders in a single patient.^37^ Our data have shown that group CBT may be an effective option for the treatment of HSDD, which may act on most of the related factors and have a positive influence on women's sexuality.

Group CBT has some advantages over the individual approach. In a group, there is the possibility of mutual identification and support among the participants, which may justify our results. Sharing of intimate information can reduce the anxiety associated with feelings of shame and secrecy as participants realize that their inadequacies are not unique;^43^ such a realization may exert a beneficial influence on women who have undergone group CBT. An additional benefit of group CBT is the absence of the side effects and risks associated with pharmacological treatment.

The association of some form of psychotherapy with pharmacological alternatives is desirable.^44^ The literature shows great variability in the type of therapeutic interventions and in the measurement of results,^45^^,^^46^ thereby hindering data interpretation. In addition, previous studies that assess the results of psychotherapeutic treatments for female sexual disorders present major challenges, including a) Clearly establishing treatment goals and parameters to be used for evaluation; b) Detailing procedures and methodology in order to allow their replication by other researchers; and c) Defining adequate and randomized control groups.^47^^,^^48^ As a result, there are few quality studies for the assessment of nonpharmacological treatments for FSD.^14^^,^^15^ In planning this study, the authors tried to take these factors into consideration. There are some well-designed studies showing the efficacy of CBT for genital pain and associated psychological domains,^35^^,^^49^, ^50^, ^51^ but not for HSDD.

Forming a control group in studies involving psychotherapeutic approaches is a hard task due to the interpersonal nature of the intervention and the difficulty in defining a masking protocol for the procedure.^23^^,^^24^ To overcome this problem, the authors used women on the waiting list as controls, as was done in other studies.^51^^,^^52^ The authors offered them an educational activity unrelated to sexuality. Nevertheless, this may be a weakness in our study. The variation of the FSQQ scores in the control group was negative or near zero, suggesting that sexual disorders rarely resolve spontaneously.

The results of the meta-analysis by Frühauf (2013) indicate that CBT could have positive effects on HSDD. However, the small number of patients in the studies and the poor research quality did not allow the drawing of definitive conclusions about the efficacy of CBT. Instead, the flaws pointed to the need for more controlled studies.^48^^,^^53^^,^^54^

Our work was an effort to contribute to the fulfillment of this need.

Group treatment has many advantages for patients. Feelings of shame and isolation are experienced by many given the difficulty to talk about sexual issues. Participation in group therapy enables the patient to realize that she is not the only person in the world to experience a sexual difficulty. Another advantage is the exchange of experiences and coping strategies related to a problem proposed by peers rather than a technical opinion given by a professional far from the patients’ biopsychosocial context.^23^ These factors may partly explain the improvement made by the women who underwent group CBT.

Group therapy fits in with the desirable characteristics for use in health services as it provides a low-cost and time-limited therapeutic option with a good clinical outcome for a large number of women.^23^^,^^24^^,^^30^ Further studies are needed to identify specific factors that may influence the outcome of the therapeutic process in order to enable primary care professionals to recognize and refer dysfunctional patients most likely to benefit from therapy.^32^ This study opens up the possibility of developing a model of short-term focal intervention carried out by a team of gynecologists who are specialized in sexual health and work in high-complexity care.

## Conclusion

Cognitive-behavioral group therapy may be effective in treating HSDD in Brazilian women. Still, further studies are needed to assess potential factors influencing treatment adherence and long-term outcome.

## Authors’ contributions

All authors contributed to the study's conception and design. Material preparation, data collection and analysis were performed by Théo Lerner, Elsa Aida Gay de Pereyra, Lucivanda Fonteles Pontes, Isabel Cristina Esposito Sorpreso and José Maria Soares Júnior. The first draft of the manuscript was written by Théo Lerner and all authors commented on previous versions of the manuscript. All authors read and approved the final manuscript.

## Funding

This study received no funding

## Ethics approval

All methods were performed in accordance with the relevant guidelines and regulations. The questionnaire and methodology for this study were approved by the Human Research Ethics committee of the Faculdade de Medicina da Universidade de São Paulo (case No. 0346/08-IRB).

## Consent to participate

Informed consent was obtained from all individual participants included in the study.

## Consent for publication

Not applicable.

## Clinical trial protocol

This study was registered in the Brazilian Clinical Trial Registry ‒ ReBEC (https://ensaiosclinicos.gov.br/) under #12087.

## Data access statement

Research data is available upon request to the corresponding author.

## Conflicts of interest

The authors declare no conflicts of interest.
